# Genomic Features and Clinical Characteristics of Adolescents and Young Adults With Cholangiocarcinoma

**DOI:** 10.3389/fonc.2019.01439

**Published:** 2020-01-14

**Authors:** Hao Feng, Huan Tong, Jiayan Yan, Min He, Wei Chen, Jian Wang

**Affiliations:** ^1^Department of Biliary-Pancreatic Surgery, School of Medicine, Renji Hospital, Shanghai Jiao Tong University, Shanghai, China; ^2^University Hospital of LMU Munich, Medical Faculty of Ludwig-Maximilians-University of Munich, Munich, Germany; ^3^Clinical and Translational Research Center, Shanghai First Maternity and Infant Hospital, Tongji University School of Medicine, Shanghai, China

**Keywords:** adolescents and young adults (AYAs), mutation, cholangiocarcinoma, early-onset, ASXL1

## Abstract

**Background:** Adolescents and young adults (AYAs) diagnosed with cancer between ages 15 and 45 years may exhibit unique biologic and genomic characteristics as well as clinical features, resulting in differences in clinical characters and drug resistance. However, compared to other solid cancers, relatively few studies have been conducted in this age group in cholangiocarcinoma (CCA). This study is performed to investigate the clinical and molecular features of AYAs with CCA.

**Methods:** Three cohorts, including the external dataset (TCGA and MSKCC) and the perihilar CCA databank of Chinese tertiary hospitals, were contained in this study. Pathway and process enrichment analysis had been carried out with the following ontology sources: KEGG Pathway, GO Biological Processes, Reactome Gene Sets, Canonical Pathways, and CORUM. Metascape and GEPIA datasets were used for bioinformatic analysis. *P* < 0.05 was considered statistically significant. All statistical analyses were performed with GraphPad Prism (version 7.0; GraphPad Software, La Jolla, California) and R studio (version 3.6.1; R studio, Boston, Massachusetts).

**Results:** Compared to older adults, AYAs with CCA presented with worse overall survival, although the difference was not significant. Specific to patients with stage IV CCAs who underwent chemotherapy, AYAs were associated with significantly poorer overall survival (OS) (*p* = 0.03, hazards ratio (HR) 3.01, 95% confidence interval (CI) 1.14-4.91). From the anatomical perspective, more extrahepatic CCA was detected in the AYA group. Microsatellite instability (MSI) occurred in 3% of older patients in the present study. Nevertheless, none of the AYAs had MSI status. In this study, AYAs gained an enhanced frequency of additional sex combs like 1 (ASXL1) (*p* = 0.02) and KMT2C (*p* = 0.02) mutation than their older counterparts. Besides ASXL1 and KMT2C, the genes enriched in AYAs with CCA were analyzed by pathway and process enrichment analysis. And those genes were found to be associated with poorer differentiation, deubiquitination, and WNT signal pathway. Moreover, AYAs were relevant to poor differentiation and advanced tumor stage.

**Conclusion:** This study offered a preliminary landscape of the clinical and molecular features of early-onset biliary cancers. Further studies including more samples are essential to investigate whether ASXL1 and KMT2C could be considered as potentially targetable genomic signatures for young patients.

## Introduction

Cholangiocarcinoma (CCA) is a highly fatal malignant tumor with rising incidence. It accounts for ~10–25% of all hepatobiliary malignancies and <1% of all types of cancers (1). The incidence of adolescents and young adults (AYAs) with CCA was even less. Despite recognition of the importance of AYAs with cancers, the biologic and genomic characteristics of AYAs with CCA remain largely unknown.

AYAs diagnosed with cancer between ages 15 and 45 years may exhibit unique biologic and genomic features, resulting in differences in clinical behaviors and chemotherapy/targeted therapy resistance (2). These features could also be clinically exploited to develop companion diagnostics and novel therapies for treating AYAs with cancers (3). For instance, AYAs with solid tumors, such as colorectal carcinomas, are more likely to exhibit signet-ring histology, synchronous or metachronous metastasis, and present at a late stage (4, 5). From the mutational perspective, most early-onset (age <50 years) patients present with lower prevalence of KRAS, BRAF, and NRAS mutations in comparison with late-onset patients (6).

To date, AYAs with other solid tumors have been extensively described in the literature. However, few studies have been conducted for patients with CCA at this age group. Despite, most recently, genomic analysis of patients with CCA being performed by the Cancer Genome Atlas (TCGA) and Memorial Sloan Kettering Cancer Center (MSKCC) (7), the genomic underpinnings of these AYAs with this rare cancer remain largely unknown. Therefore, in this study, the clinical and molecular features of AYA CCA patients were investigated by analyzing the external dataset (8, 9) and internal hilar CCA databank to shed light on early-onset biliary malignancy.

## Methods

### Study Population and Data Collection

Three cohorts were included in the present study. The first cohort included 155 consecutive patients with perihilar CCA (pCCA) from three hepatobiliary surgery centers affiliated to tertiary hospitals in China between January 2013 and November 2018. Eighteen patients (12%) in this cohort were AYA (aged 15–45 years) and were set as *AYA group*. The rest (age >45) was set as the group “*Others*.” This retrospective study was approved by the institutional review board (IRB) of the Renji Hospital and the Study Group of Biliary Surgery of the surgical branch of the Chinese Medical Association.

In the second cohort, the genomic data (e.g., mutation frequency) of AYAs and the elderly with CCAs extracted from the TCGA database were compared. This cohort included five AYA (10%) and 46 elder patients.

The third cohort contained the data of age-associated gene mutation of 192 patients with CCA extracted from the MSKCC dataset, including 26 (14%) AYAs. cBioPortal platform (www.cbioportal.org) was used for analyzing (8, 9) (Table 1).

**Table 1:** The clinical character of AYA patients and older patients.

	**AYA (<=45)**		**Others (>45)**	
**Sample size**	26		166	
**Gender (male:female)**	12:14		88:78	
Age (year)*	40 (26–45)		64 (46–87)	
**Stage IV**	15	58%	112	68%
**Recurrence**				
Recurrence	6	23%	36	22%
Non-recurrence	4	15%	4	2%
Not applicable	17	65%	125	75%
**Metastatic site**	7	27%	43	26%
Liver	3	12%	12	7%
Lung	0	0%	4	2%
Lymph node	0	0%	13	8%
Brain	1	4%	0	0%
Omentum	0	0%	3	2%
Peritoneum	1	4%	6	4%
Pleura	2	8%	0	0%
Pelvis	0	0%	1	1%
Others	0	0%	4	2%
**MSI score**	0.88	(0–5.11)	0.94	(0–35.01)
**TMB score**	4.84	(2–17.7)	4.26	(1–47.2)
**Systematic therapy**	18	69%	138	83%
FOLFOX	2	11%	7	5%
FOLFIRINOX	2	11%	0	0%
Gemcitabine	2	11%	3	2%
Gem/cis	10	56%	64	46%
GemOX	2	11%	22	16%
Bevacizumab/FUDR	0	0%	1	1%
Cape/OX	0	0%	1	1%
Capecitabine	0	0%	1	1%
FUDR/GemOX	0	0%	9	7%
Gax	0	0%	1	1%
Gem/abraxane	0	0%	2	1%
Gem/Cape	0	0%	3	2%
Gem/Cis/MEK162	0	0%	18	13%
Gem/erlotinib	0	0%	1	1%
Gem/taxol	0	0%	1	1%
G-FLIP	0	0%	1	1%
Irinotecan + HAI FUDR	0	0%	1	1%
Sorafenib	0	0%	1	1%
TDM-1	0	0%	1	1%

* p < 0.05;

** *p < 0.01*.

### Follow-Up

In the present study, progression-free survival (PFS) was defined as the time after the treatment with the disease not getting worse. Disease-free survival (DFS) was the time for any recurrence. If the postoperative margin was negative, the operation was considered as R_0_ resection. Follow-up consisted of serum tumor marker measurements every 1–3 months and computed tomography (CT) every 6 months. Complete follow-up was conducted for the entire cohort of patients.

### Pathological Evaluation

Tumor specimens were sent for pathological evaluation about the quality, grading, tumor stage according to AJCC 7th edition, risk factor (perineuronal invasion, etc.), and lymph node status. CCAs are a heterogeneous group of tumors that can be classified into three clinically distinct types of cancers, intrahepatic CCA (iCCA), pCCA, and distal CCA (dCCA) basing on its anatomical location. pCCA and dCCA were also grouped as extrahepatic CCA (eCCA). Specifically, pCCA in the present study was defined as the CCA that developed at the point where the left and right hepatic ducts joined to form the common hepatic duct by imaging (CT or magnetic resonance cholangiopancreatography).

### MSI/MSS Status and TMB Evaluation

Programmed cell death protein 1 (PD-1) blockade provides a therapeutic opportunity for patients with high tumor mutation burden (TMB), high microsatellite instability (10) (MSI-H), and deficient mismatch repair (dMMR). Therefore, the MSI score, microsatellite instability (MSI)/microsatellite stability (MSS) status, and TMB were also analyzed between the two groups by using cBioPortal platform.

### Perioperative Evaluation

The intraoperative evaluation included the length of operation, intraoperative hemorrhage, intraoperative blood transfusion, and vascular anastomosis. Additionally, blood routine examination, biochemical test, total bilirubin (Blood) (TBil), aspartate transaminase (AST), alanine transaminase (ALT), and so on, and other hepatic and renal function examinations were performed perioperatively.

### Enrichment Analyses

Metascape (http://metascape.org/gp/index.html) is an effective and efficient tool for experimental biologists to comprehensively analyze and interpret OMICs-based studies in the big data era (19). The database was used to perform the Gene Ontology (GO) and Kyoto Encyclopedia of Genes and Genomes (KEGG) pathway enrichment analysis, which is used to predict the potential biological functions of the overlapping genes of the DEGs and target genes. Then, verification was performed by the GEPIA database (http://gepia.cancer-pku.cn) to identify hub genes (11–19).

### Statistical Analysis

Pearson's Chi-square test for categorical variables and the Wilcoxon rank-sum test for continuous variables were used to compare various parameters in AYA and the other group. The Kaplan-Meier method was used to estimate overall survival (OS), DFS, or PFS. Differences in survival outcomes were assessed by the log-rank test. Results were presented as hazard ratios (HRs) and 95% confidence intervals (CIs). *P* < 0.05 was considered statistically significant. All statistical analyses were performed with GraphPad Prism (version 7.0; GraphPad Software, La Jolla, California) and R studio (version 3.6.1; R studio, Boston, Massachusetts).

## Results

### Clinicopathologic Features of AYAs With CCA

**Figure 1:**
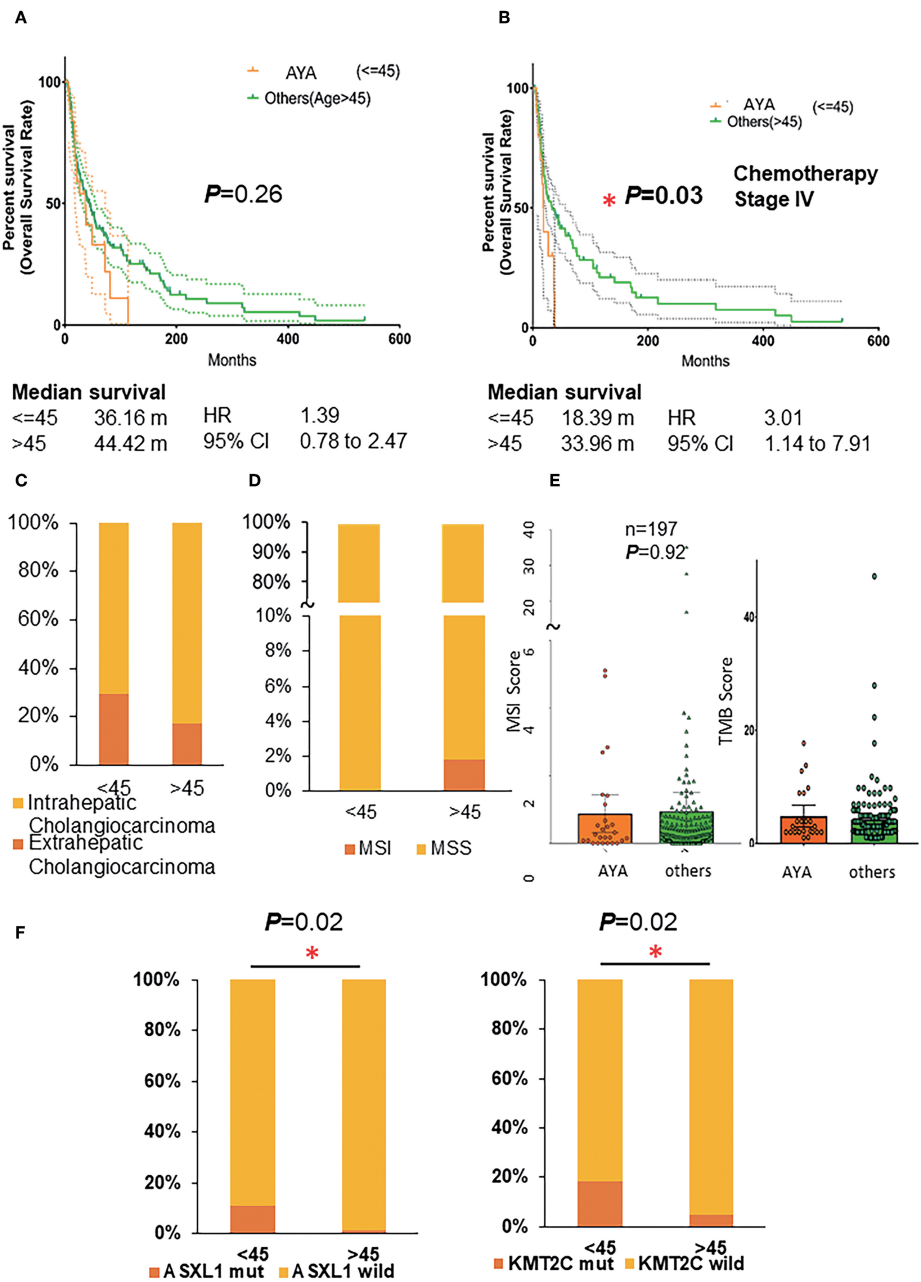
**(A)** Overall survival rate of AYA patients and others (age >45). **(B)** Overall survival rate of AYA patients and others (age >45) with stage IV cholangiocarcinoma and underwent the treatment of chemotherapy. **(C)** The proportion of intrahepatic and extrahepatic cholangiocarcinoma in AYA (<=45) and other (>45) groups. **(D)** The MSI/MSS status of patients in AYA (<=45) and other(>45) groups. **(E)** The MSI score and TMB score of patients in AYA (<=45) and other (>45) groups. **(F)** The mutation frequency of ASXL1 and KMT2C of patients in AYA (<=45) and other (>45) groups basing on cohort 3 (MSKCC). AYA, adolescents and young adults; MSI, microsatellite instability; TMB, tumor mutation burden.

From the prognosis perspective, the length of OS in AYAs with CCA was worse (36 vs. 44 months) than the older patients. However, the difference was not significant (Figure 1A; *p* = 0.26, HR 1.39, 95% CI 0.78–2.47). Specific to patients with stage IV CCAs who underwent chemotherapy, AYAs were associated with significantly poorer OS (Figure 1B; *p* = 0.03, HR 3.01, 95% CI 1.14–4.91), and the survival period was almost half of their older counterparts (18 vs. 34 months). From the anatomical perspective, more eCCA was detected in the AYA group (29 vs. 17%, Figure 1C).

### Molecular Features of AYAs With CCA

PD-1 blockade provides a therapeutic opportunity for patients with high TMB, MSI-H, and dMMR. Therefore, the MSI score, MSI/MSS status, and TMB (Figure 1D) were also analyzed between the two groups. It has been reported that MSI status occurred in 3–10% of CCA; consistently, MSI occurred in 3% of older patients (>45 years old) in the present study. Intriguingly, none of the AYA patients had MSI status, although the average MSI score was similar (Figure 1E; AYA group: 0.8785 ± 0.2727, Others group: 0.944 ± 0.2831) between the two groups. Additionally, AYA patients had similar TMB compared to their counterparts (AYA group: 4.258 ± 0.3885, Others group: 4.452 ± 0.8883).

### Somatic Mutations of CCA in AYA Patients

Additional sex combs like 1 (ASXL1) is the obligate regulatory subunit of a deubiquitinase complex. Heterozygous mutations of ASXL1 are frequent in myeloid leukemias and other malignancies. Here we demonstrated in the first cohort that AYAs with CCAs gained a higher frequency of ASXL1 mutation than their older counterparts [Figure 1F; *p* = 0.02, 11% (3/27) vs. 1% (2/167)].

KMT2C mutates frequently and is considered crucial for the occurrence and development of numerous cancers. In the present study, significantly higher KMT2C (histone lysine methyltransferase 2C) mutation rate was in the AYA group [Figure 1F; *p* = 0.02, 19% (5/27) vs. 4.7% (8/169)]. Specifically, 40% of the patients who had mutated ASXL1 also harbored a mutated KMT2C (also known as MLL3), KMT2D, or ARID1A. And 38.5% of the KMT2C mutated synergistically with ARID1A mutation. Additionally, although the difference was not significant, AYAs were likely to harbor more frequent mutated FGFR2 (18.5 vs. 9.5%) or PBRM1 (18.5 vs. 9.5%) or ERBB3 (11.1 vs. 2.4%) genes and less BAP1, KRAS, and SMAD4 (Supplemental Figures 1A,B; Table 2).

**Table 2:** Comparison of genes with different mutation frequency in both groups.

**Gene**	**Cytoband**	**(A) AYA**	**(B) OA**	***p*-value**	**Gene**	**Cytoband**	**(A) AYA**	**(B) OA**	***p*-value**
ASXL1	20q11.21	3 (11.11%)	2 (1.19%)	0.0197	EP300	22q13.2	1 (3.70%)	1 (0.60%)	0.258
KMT2C	7q36.1	5 (18.52%)	8 (4.76%)	0.0206	ERBB4	2q34	1 (3.70%)	1 (0.60%)	0.258
ERBB3	12q13.2	3 (11.11%)	4 (2.38%)	0.0569	FLT4	5q35.3	1 (3.70%)	1 (0.60%)	0.258
FAT1	4q35.2	2 (7.41%)	2 (1.19%)	0.093	FOXA1	14q21.1	1 (3.70%)	1 (0.60%)	0.258
SOX9	17q24.3	2 (7.41%)	2 (1.19%)	0.093	KIAA1217	10p12.2-p12.1	1 (3.70%)	1 (0.60%)	0.258
KRAS	12p12.1	1 (3.70%)	22 (13.10%)	0.136	MALT1	18q21.32	1 (3.70%)	1 (0.60%)	0.258
AR	Xq12	1 (3.70%)	0 (0.00%)	0.138	MEN1	11q13.1	1 (3.70%)	1 (0.60%)	0.258
AXIN2	17q24.1	1 (3.70%)	0 (0.00%)	0.138	MST1R	3p21.31	1 (3.70%)	1 (0.60%)	0.258
CDKN1A	6p21.2	1 (3.70%)	0 (0.00%)	0.138	NCOA3	20q13.12	1 (3.70%)	1 (0.60%)	0.258
DICER1	14q32.13	1 (3.70%)	0 (0.00%)	0.138	TCF3	19p13.3	1 (3.70%)	1 (0.60%)	0.258
FGFR4	5q35.2	1 (3.70%)	0 (0.00%)	0.138	TSC2	16p13.3	1 (3.70%)	1 (0.60%)	0.258
GATA2	3q21.3	1 (3.70%)	0 (0.00%)	0.138	ZFHX3	16q22.2-q22.3	1 (3.70%)	1 (0.60%)	0.258
GNA11	19p13.3	1 (3.70%)	0 (0.00%)	0.138	PIK3CA	3q26.32	3 (11.11%)	10 (5.95%)	0.261
GRIN2A	16p13.2	1 (3.70%)	0 (0.00%)	0.138	BAP1	3p21.1	2 (7.41%)	24 (14.29%)	0.262
HIST3H3	1q42.13	1 (3.70%)	0 (0.00%)	0.138	TERT	5p15.33	0 (0.00%)	8 (4.76%)	0.296
JAK1	1p31.3	1 (3.70%)	0 (0.00%)	0.138	TP53	17p13.1	8 (29.63%)	39 (23.21%)	0.307
LAMC1	1q25.3	1 (3.70%)	0 (0.00%)	0.138	SMAD4	18q21.2	1 (3.70%)	15 (8.93%)	0.317
MDM2	12q15	1 (3.70%)	0 (0.00%)	0.138	ATM	11q22.3	1 (3.70%)	14 (8.33%)	0.354
NOL4	18q12.1	1 (3.70%)	0 (0.00%)	0.138	KMT2D	12q13.12	2 (7.41%)	7 (4.17%)	0.361
PDCD1	2q37.3	1 (3.70%)	0 (0.00%)	0.138	APC	5q22.2	1 (3.70%)	2 (1.19%)	0.362
PHOX2B	4p13	1 (3.70%)	0 (0.00%)	0.138	ATR	3q23	1 (3.70%)	2 (1.19%)	0.362
PLK2	5q11.2	1 (3.70%)	0 (0.00%)	0.138	BRD4	19p13.12	1 (3.70%)	2 (1.19%)	0.362
RABGAP1L	1q25.1	1 (3.70%)	0 (0.00%)	0.138	GATA1	Xp11.23	1 (3.70%)	2 (1.19%)	0.362
RASAL2	1q25.2	1 (3.70%)	0 (0.00%)	0.138	IGF1R	15q26.3	1 (3.70%)	2 (1.19%)	0.362
SOX17	8q11.23	1 (3.70%)	0 (0.00%)	0.138	KDM5A	12p13.33	1 (3.70%)	2 (1.19%)	0.362
STAG2	Xq25	1 (3.70%)	0 (0.00%)	0.138	PTCH1	9q22.32	1 (3.70%)	2 (1.19%)	0.362
TACC2	10q26.13	1 (3.70%)	0 (0.00%)	0.138	XPO1	2p15	1 (3.70%)	2 (1.19%)	0.362
TGFBR2	3p24.1	1 (3.70%)	0 (0.00%)	0.138	DOT1L	19p13.3	0 (0.00%)	6 (3.57%)	0.404
ARID1B	6q25.3	2 (7.41%)	3 (1.79%)	0.142	CDH1	16q22.1	1 (3.70%)	3 (1.79%)	0.452
CTNNB1	3p22.1	2 (7.41%)	3 (1.79%)	0.142	EPHA5	4q13.1-q13.2	1 (3.70%)	3 (1.79%)	0.452
KMT2A	11q23.3	2 (7.41%)	3 (1.79%)	0.142	IDH2	15q26.1	1 (3.70%)	3 (1.79%)	0.452
FGFR2	10q26.13	5 (18.52%)	16 (9.52%)	0.144	MAP2K1	15q22.31	1 (3.70%)	3 (1.79%)	0.452
PBRM1	3p21.1	5 (18.52%)	16 (9.52%)	0.144	MAP3K1	5q11.2	1 (3.70%)	3 (1.79%)	0.452
SMARCA4	19p13.2	2 (7.41%)	4 (2.38%)	0.195	POLE	12q24.33	1 (3.70%)	3 (1.79%)	0.452
NRAS	1p13.2	2 (7.41%)	5 (2.98%)	0.25	SETD2	3p21.31	1 (3.70%)	3 (1.79%)	0.452
ASXL2	2p23.3	1 (3.70%)	1 (0.60%)	0.258	ARID2	12q12	0 (0.00%)	5 (2.98%)	0.471
CARD11	7p22.2	1 (3.70%)	1 (0.60%)	0.258	ARID1A	1p36.11	5 (18.52%)	35 (20.83%)	0.507
CREBBP	16p13.3	1 (3.70%)	1 (0.60%)	0.258	IDH1	2q34	7 (25.93%)	41 (24.40%)	0.516
EIF4A2	3q27.3	1 (3.70%)	1 (0.60%)	0.258					

**Figure 2:**
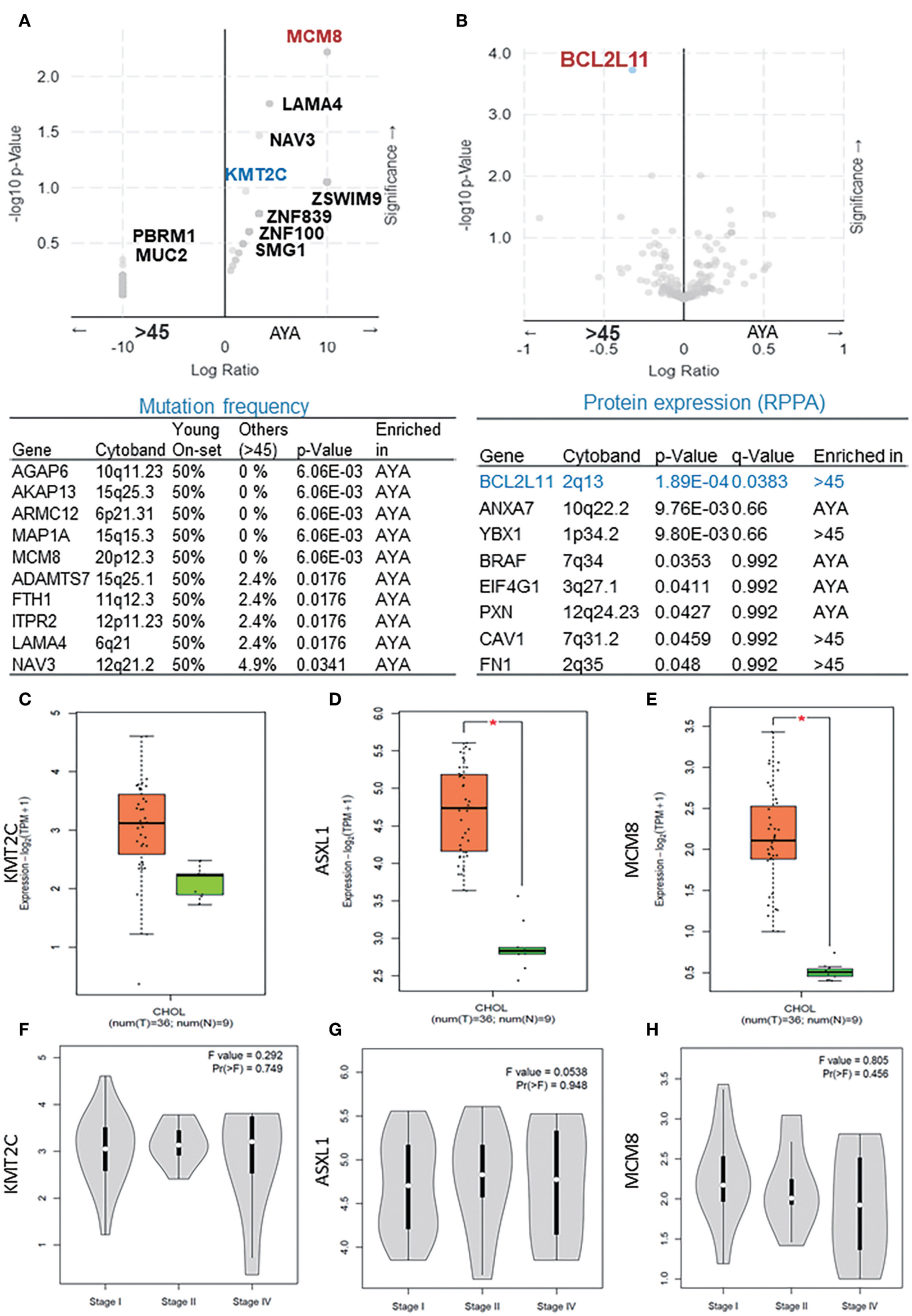
**(A)** The mutation frequency of presentative genes (*p* < 0.05) in AYA and other groups basing on cohort 2. **(B)** The difference of protein expression between the two groups basing on cohort 2. **(C–E)** The expression level of ASXL1, KMT2C, and MCM8 in tumor vs. paired normal samples in CCA. **(F–H)** Expression level of ASXL1, KMT2C, and MCM8 in different tumor stages. AYA, adolescents and young adults; ASXL1, additional sex combs like 1. *P < 0.05.

In the second cohort extracted from the TCGA dataset, the MCM8 gene mutation (*p* < 0.05) was significantly enriched in AYAs with CCA. Besides KMT2C, mutations of LAMA4, AGAP6, AKAP13, ARMC12, MAP1A, NAV3, ADAMTS7, FTH1, and ITPR2 were also observed in AYAs with CCA (Figure 2A). From the protein expression aspect, BCL2L11 was significantly downregulated in AYAs (Figure 2B; *q* = 0.0383). From the RNA expression perspective, PIK3C3, IQCH, RGP1, and LPP were upregulated in the AYA group (Supplemental Figure 1C).

### Overexpression of KMT2C and ASXL1 in CCA

**Figure 3:**
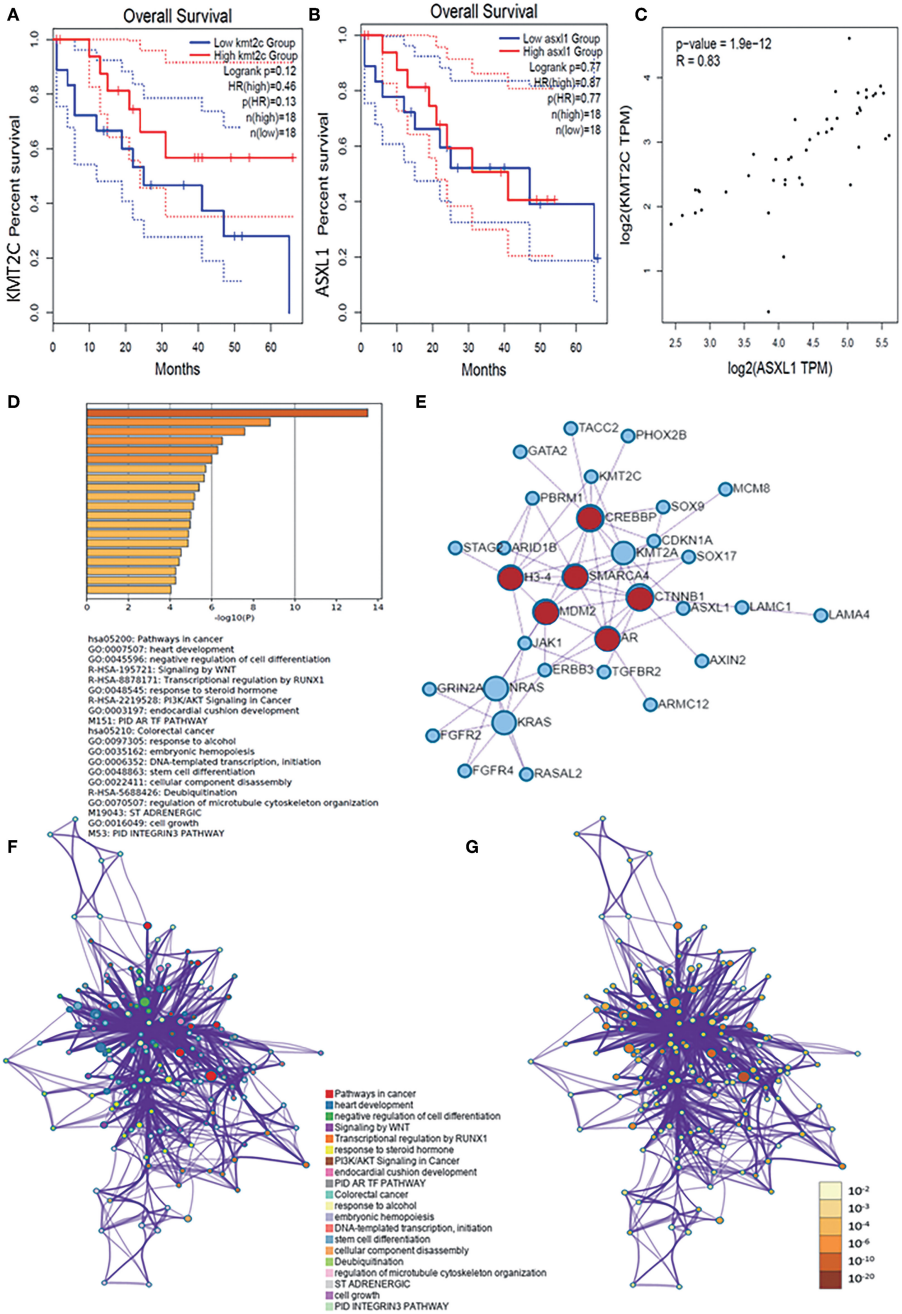
**(A,B)** Survival analysis based on the expression status of KMT2C and ASXL1 and a Kaplan-Meier curve was plotted. **(C)** Correlations of KMT2C and ASXL1 in CCA. **(D)** Bar graph of enriched terms across these enriched genes in AYAs with CCA, colored by *p*-values. **(E)** Protein–protein interaction network and MCODE components identified in the genes enriched in AYAs with CCA. **(F,G)** Network of enriched terms: **(F)** colored by cluster-ID, where nodes that share the same cluster ID are typically close to each other; **(G)** colored by *p*-value, where terms containing more genes tend to have a more significant *p*-value. ASXL1, additional sex combs like 1; AYA, adolescents and young adults; CCA, cholangiocarcinoma.

We then verified the expression level of KMT2C, ASXL1, and MCM8 in CCA using the GEPIA database and found that all of the three genes, especially ASXL1 (*p* < 0.05) and MCM8 (*p* < 0.05), were overexpressed in tumor tissues (Figures 2C–E). However, the expression level of the three genes was associated with neither tumor stages nor OS rate, respectively (Figures 2F–H, 3A,B). Pearson's correlation coefficient of ASXL1 and KMT2C was 0.83 (Figure 3C).

### Pathway and Process Enrichment Analysis of the Enriched Genes in AYAs

For these enriched genes in AYAs with CCA, pathway and process enrichment analysis had been carried out with the following ontology sources: KEGG Pathway, GO Biological Processes, Reactome Gene Sets, Canonical Pathways, and CORUM. Top 20 clusters with their enriched representative terms were shown in Figure 3D. To further capture the relationships between the terms, a subset of enriched terms had been selected and rendered as a network plot, where terms with a similarity >0.3 were connected by edges. The network was visualized using Cytoscape, where each node represented an enriched term and was colored first by its cluster ID (Figure 3F) and then by its *p*-value (Figure 3G). Specifically, the genes enriched in AYAs with CCA were associated with several pathways, such as cancer-associated pathways, negative regulation of cell differentiation, deubiquitination, WNT signal pathway, and so on.

Then, for these enriched genes in AYAs with CCA, protein–protein interaction enrichment analysis had also been carried out. Densely connected network components, including MDM2, SMARCA4, CTNNB1, AR, CREBBP, H3-4, were identified in Figure 3E.

### Clinical Characters and Postoperative Prognosis of AYAs With pCCA

**Figure 4:**
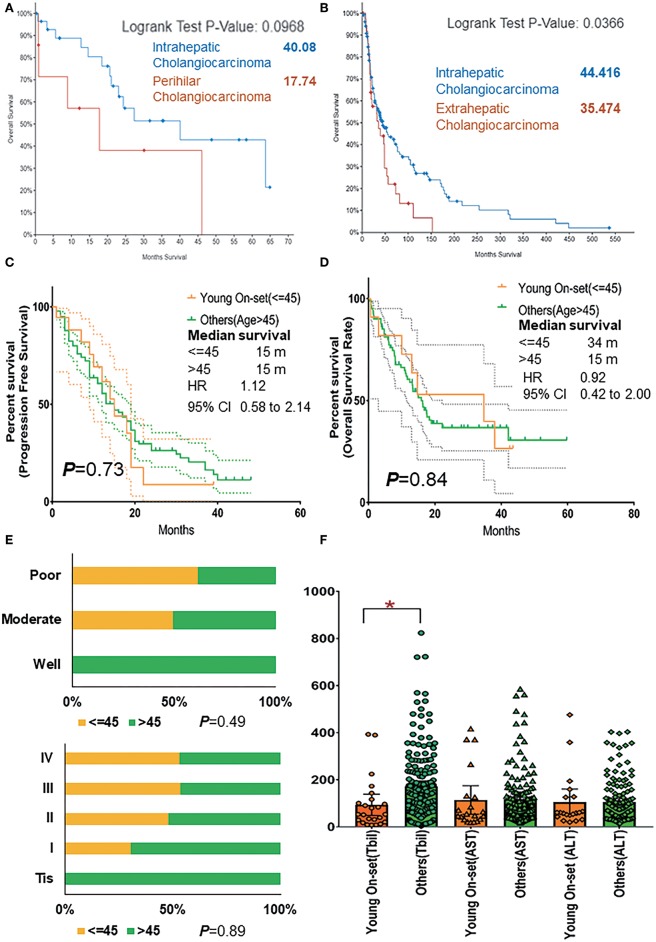
**(A)** Comparison of overall survival rate of patients with intrahepatic and perihilar CCA basing on cohorts 2 and 3. **(B)** Comparison of overall survival rate of patients with intrahepatic and extrahepatic CCA basing on cohorts 2 and 3. **(C)** The progression-free survival rate of AYAs and others (age >45) with pCCA basing on cohort 1. **(D)** The overall survival rate of AYAs and others (age >45) with pCCA basing on cohort 1. **(E)** The proportion and ratio of different grades of differentiation and different pathological stages in AYA and others (age >45) group. **(F)** The comparison of TBil, AST, and ALT expression in AYA (<=45, young-onset) group and other (>45) group. ALT, alanine transaminase; AST, aspartate transaminase; AYA, adolescents and young adults; CCA, cholangiocarcinoma; pCCA, perihilar CCA; TBil, total bilirubin (blood). *P < 0.05.

External genomic profiles (cohort 2, cohort 3) were analyzed, and it was found that iCCA presented significant better OS than eCCA (*p* = 0.04, 44 vs. 35 months) and slightly better than pCCA, too (*p* = 0.09, 40 vs. 18 months) (Figures 4A,B).

As is known, for patients in the intrahepatic, perihilar, and distal groups, the 5-year survival was 40, 10, and 23%, respectively (20). The prognosis of pCCA was the worst. Thus, by using our pCCA dataset containing 245 patients, we further investigated the prognosis between AYAs (cohort 1) and older patients (>45). Intriguingly, these patients had similar PFS (Figure 4C; *p* = 0.73, 15 vs. 15 months, HR 1.12, 95% CI 0.58–2.14) and OS rate (Figure 4D; *p* = 0.84, 34 vs. 15 months, HR 0.92, 95% CI 0.42–2.00).

Moreover, it was shown that AYAs were relevant to poor differentiation (Figure 4E) and advanced tumor stage (III and IV, 67%, Figure 4E). All AYAs in the current study presented with moderate and poor differentiation (Table 1). The comparison of chemical examinations showed that TBil value of older patients (>45 years old) were significantly elevated (Figure 4F).

## Discussion

Recognition of the clinical and genomic characters of AYAs with CCA is crucial for treatment strategy design. The treatments, especially targeted therapy and immunotherapy of AYAs, may differ from those best suited to older patients. It was reported that solid cancers (21), such as colorectal carcinoma, in AYAs were more aggressive and associated with a poorer prognosis as well as enriched MSI-H status compared to older patients (22, 23). In contrast, no MSI status was detected in AYAs with CCA in the present study. In the older patients' group, MSI occurred in 3~10% of the patients, similar to the reported general probability in all CCAs. The length of survival of AYAs (1.5 years) was almost half of the older patients (3 years); however, owing to the small sample size, no statistical significance was achieved. This was also the limitation of the present study.

The present study provided an initial landscape of genes that displays a greater mutational frequency in AYAs with CCA. Specifically, ASXL1 and KMT2C were found more frequent in AYAs compared with older patients with CCA.

ASXL1 mutations were known to be upregulated in solid cancers with metastasis (24) and in castration-resistant prostate cancer (CRPC) (25). Intriguingly, the significantly greater mutation frequency of ASXL1 combined with lower KRAS mutation was reported in kinase rearrangements (KRE). And lower KRAS mutation frequency was also detected in AYA patients as reported. Moreover, the high mutation rate of ASXL1 rates was also associated with MSI status enrichment (26). In the present study, the mutation frequency of ASXL1 was significantly higher in AYAs. KRAS mutation also tended to decrease but without statistical significance owing to the inadequate sample size. The only inconsistency was that all AYAs with CCA had MSS status instead of MSI. Patients with MSI-H status and KRE could benefit from both tyrosine kinase inhibitor (TKI) and checkpoint inhibitor treatment. However, this advantage seems to attenuate in AYAs with CCA. In contrast, it was reported that the transcription regulator ASXL1 mutation was associated with poorer outcomes as well as drug resistance (27), which might explain why AYAs with stage IV CCAs who underwent chemotherapy had worse prognosis in the present study.

Similar to ASXL1, KMT2C mutation was also enriched in late-stage or metastatic status of iCCA (28), breast cancer (29), and prostate cancer (30) and was associated to poor prognosis (31). Especially in AYAs with late-stage CCA, greater ASXL1 and KMT2C mutation rates were detected, which might suggest that CCAs in AYA patients is more aggressive.

Besides ASXL and KMT2C, the genes enriched in AYAs with CCA were analyzed by pathway and process enrichment analysis. And those genes were found to be associated with poorer differentiation, deubiquitination, and WNT signal pathway. Surgical resection remains the mainstay of potentially curative treatment for CCA. However, the probability of radical curative resection is low, and the prognosis is insufficient. Molecular profiling has delineated the genomic and transcriptomic characters of each CCA subtype. However, the genomic signature of AYA patients was not reported before. This study offered a preliminary landscape of the clinical and molecular features of early-onset biliary cancers. Further studies including more samples are essential to investigate whether ASXL1 and KMT2C could be considered potentially targetable genomic signatures for young patients.

## Data Availability Statement

The raw data supporting the conclusions of this manuscript will be made available by the authors, without undue reservation, to any qualified researcher.

## Ethics Statement

The studies involving human participants were reviewed and approved by Institutional Review Board (IRB) of the Renji Hospital and Study Group of Biliary Surgery of the surgical branch of the Chinese Medical Association. The patients/participants provided their written informed consent to participate in this study.

## Author Contributions

HF and JW conceived the project, designed the study, and drafted the manuscript. HF and HT designed the study, wrote and revised the manuscript, and approved the final submission. HF, HT, and JW revised the manuscript and approved the final submission. JY, WC, and MH were involved in the design of the study. All authors read and approved the manuscript. All authors qualify as per ICJME criteria for authorship.

### Conflict of Interest

The authors declare that the research was conducted in the absence of any commercial or financial relationships that could be construed as a potential conflict of interest.

## Supplementary Material

Supplemental Figure 1 **(A)** The mutation frequency of FGFR2, PBRM1, ERBB3 elevated in patients of AYA (< = 45) group; The mutation frequency of BAP1, KRAS, SMAD4 elevated in patients of others (>45) groups basing on cohort 3 (MSKCC); **(B)** A summary of presentative mutation in AYA and other groups basing on cohort 3 (MSKCC). AYA, Adolescents, and young adults; mut, mutation. **(C)** The difference of RNA expression between the two groups basing on cohort 2.
