# Supplementary figures and images for: Genomic Features and Clinical Characteristics of Adolescents and Young Adults With Cholangiocarcinoma
Source: Front Oncol. 2020 Jan 14;9:1439. doi: 10.3389/fonc.2019.01439 (PMC6971196; doi:10.3389/fonc.2019.01439)

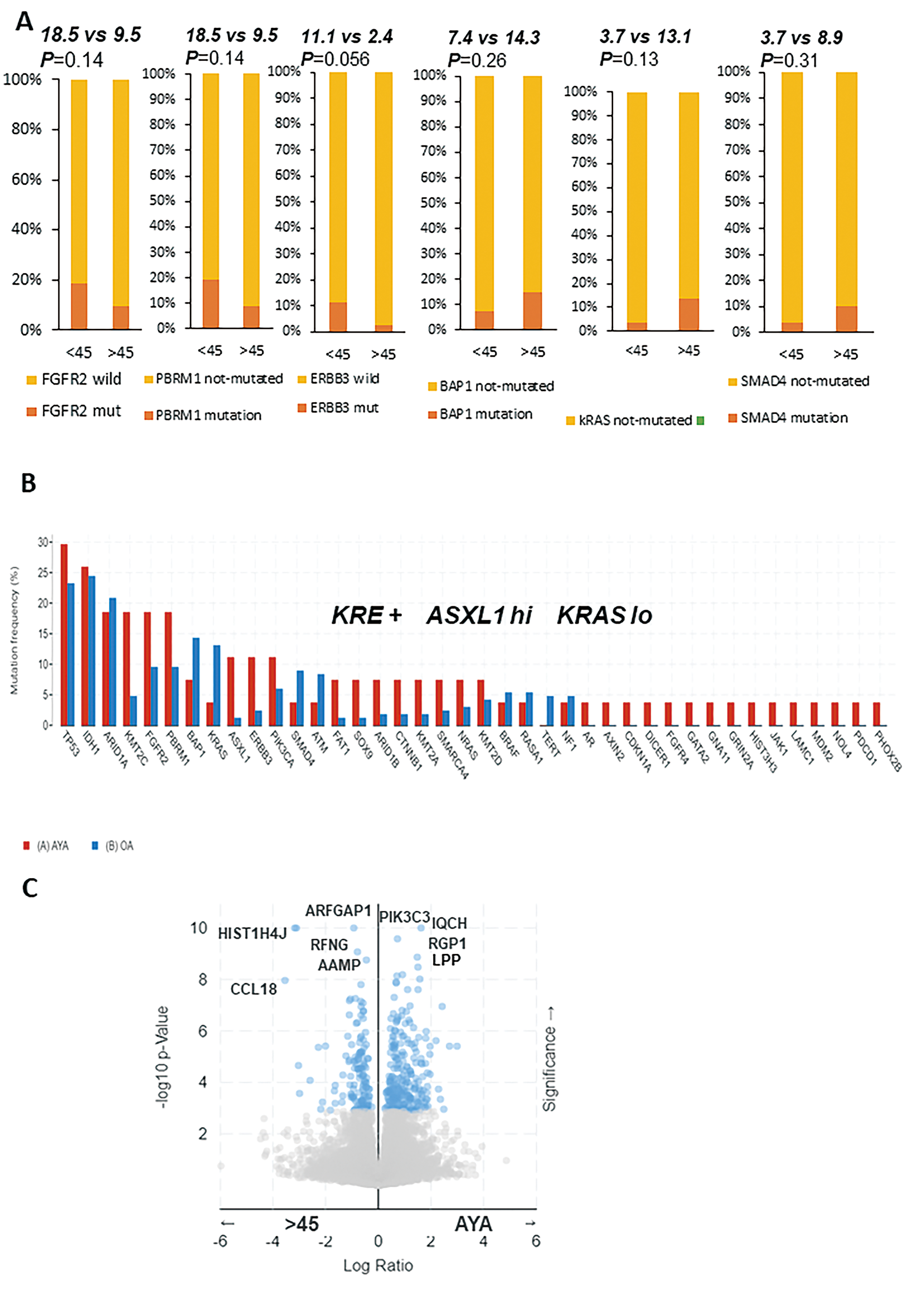

Supplement: Supplemental Figure 1 — (A) The mutation frequency of FGFR2, PBRM1, ERBB3 elevated in patients of AYA (< = 45) group; The mutation frequency of BAP1, KRAS, SMAD4 elevated in patients of others (>45) groups basing on cohort 3 (MSKCC); (B) A summary of presentative mutation in AYA and other groups basing on cohort 3 (MSKCC). AYA, Adolescents, and young adults; mut, mutation. (C) The difference of RNA expression between the two groups basing on cohort 2. [file Image_1.tif]
